# Are the Follicular Fluid Characteristics of Recovered Coronavirus Disease 2019 Patients Different From Those of Vaccinated Women Approaching *in vitro* Fertilization?

**DOI:** 10.3389/fphys.2022.840109

**Published:** 2022-02-22

**Authors:** Maria A. Castiglione Morelli, Assunta Iuliano, Sergio C. A. Schettini, Angela Ferri, Paola Colucci, Licia Viggiani, Ilenia Matera, Angela Ostuni

**Affiliations:** ^1^Department of Sciences, University of Basilicata, Potenza, Italy; ^2^Center for Reproductive Medicine of “San Carlo” Hospital, Potenza, Italy

**Keywords:** SARS-CoV-2, COVID-19, follicular fluid, metabolomics, NMR, anti SARS-CoV-2 immunoglobulins, proinflammatory markers

## Abstract

The aim of this pilot study is to evaluate if SARS-CoV-2 infection or vaccination against SARS-CoV-2 infection induce observable metabolic effects in follicular fluid of women who are following *in vitro* fertilization (IVF) treatments. The possible impact of coronavirus disease 2019 (COVID-19) on fertility and IVF outcome is considered. We have selected for this study: six women vaccinated against SARS-CoV-2 infection, five recovered COVID-19 patients, and we used nine healthy women as the control group. At the time of oocytes retrieval from participants in the study, follicular fluids were collected and metabolomic analysis was performed by ^1^H NMR spectroscopy in combination with multivariate analysis to interpret the spectral data. The search for antibody positivity in the follicular fluid aspirates was also carried out, together with the western blotting analysis of some inflammatory proteins, interleukin-6, tumor necrosis factor α (TNFα), and the free radical scavenger superoxide dismutase 2. Higher levels of Ala and Pro together with lower levels of lipids and trimethylamine N-oxide (TMAO) were found in follicular fluids (FFs) of vaccinated women while lower levels of many metabolites were detected in FFs of recovered COVID patients. Expression level of TNF-α was significantly lower both in recovered COVID-19 patients and vaccinated women in comparison to healthy controls.

## Introduction

After a nearly complete pause at the beginning of the coronavirus disease 2019 (COVID-19) pandemic (Alviggi et al., 2020; Andrabi et al., 2020), assisted reproductive technology (ART) treatments restarted with proper screening and testing of the patients and safety precautions (Rajput et al., 2021a,b). With pandemic, new questions arose in all fields of medicine and healthcare and also in the context of *in vitro* fertilization (IVF) treatments, where at the moment there are not enough data on the possible effects of SARS-CoV-2 on fertility and IVF outcome (Madjunkov et al., 2020; Elsaddig and Khalil, 2021), and also on the indirect impact of COVID-19 on reproductive medicine (Kassie et al., 2021).

It has been documented the absence of viral RNA in follicular fluid (FF) of a SARS-CoV-2-positive woman and this suggests that handling of oocytes, sperm, seminal fluid, or follicular fluid in IVF practice does not constitute a significant source of infection (Demirel et al., 2021). Also, the Belgian COVART study showed that SARS-CoV-2 RNA was undetectable in follicular fluid or vaginal secretions in the cohort of women analyzed (Kteily et al., 2021). A recent work by Israeli researchers indicates that specific antibodies to the virus are present in both serum and ovarian follicles following infection or vaccination, and no apparent adverse effect on follicular function is observed (Bentov et al., 2021).

Many of the questions related to the metabolic pathway of COVID-19 disease remain to be addressed while the literature is still scarce and the infection resulting from SARS-CoV-2 is not yet fully understood at the cellular metabolite level. Various studies have been performed in the last months on serum (Bruzzone et al., 2020; Shen et al., 2020; Doğan et al., 2021; Páez-Franco et al., 2021; Shi et al., 2021), plasma (Chen et al., 2020; Fraser et al., 2020; Kimhofer et al., 2020; Wu et al., 2020; Lodge et al., 2021), and saliva (Costa Dos Santos Junior et al., 2020; Sapkota et al., 2020) of COVID-19 patients. The objectives of metabolomic works are to identify biomarkers with diagnostic potential in addition to give a description of the metabolic pathways involved in the different disorders observed in COVID-19 patients.

As far as we know, up to now no metabolomic study has been performed on FF of women infected by SARS-CoV-2 or vaccinated. The aim of the present study is to investigate whether there is a specific metabolic profile associated with a past COVID-19 positivity or vaccination in FF of women who are following IVF treatments; as a control we used FF collected before pandemic. Nuclear magnetic resonance spectroscopy (NMR) has shown its utility in various stages of ART (Asampille et al., 2020). In this study we have used NMR-based metabolomics that was shown to be well suited for the characterization of FF and to quantify and identify its metabolites (Pñero-Sagredo et al., 2010; Baskind et al., 2011; McRae et al., 2012; Wallace et al., 2012; O’Gorman et al., 2013).

Follicular fluid is an important component in the growth and development of the follicle which consists of many substances secreted from granulosa- and theca cells and transudates from blood compartment, such as hormones, growth factors, immune cells, cytokines, enzymes, anticoagulants, electrolytes, reactive oxygen species, lipids, cholesterol, and antioxidants. Therefore, changes in FF will influence the developing oocyte (Freitas et al., 2017). Cytokines in FF are important for reproduction as they modulate oocyte maturation and ovulation which influence subsequent fertilization, development of early embryo, and potential for implantation (Field et al., 2014; Bianchi et al., 2016; Robertson et al., 2018). It has been widely reported that patients with severe COVID-19 disease may undergo the so-called cytokine storm, which recalls the role of the immune system in producing an aberrant systemic inflammatory response (Chen et al., 2021). In particular, the plasma levels of some inflammatory proteins including tumor necrosis factor (TNF) and interleukin-6 (IL-6) were significantly increased in patients with severe COVID-19 (Coperchini et al., 2021). Consequently, changes in plasma composition might be also mirrored in FF composition. Therefore, we evaluated in FF of vaccinated or immunized women the possible variation in the expression levels of two proinflammatory cytokines, TNF-α and IL-6, and superoxide dismutase 2 (SOD-2), a mitochondrial isoenzyme that convert the pro-oxidant superoxide into hydrogen peroxide. Furthermore, considering that some cytokines modulate the glucose and lipid metabolism (Shy et al., 2019), possible relationships between the expression levels of these cytokines and the presence of some metabolites could be found.

## Materials and Methods

### Study Participants

Twenty women undergoing treatment for IVF at Center for Reproductive Medicine of “San Carlo” Hospital were involved in this study. Patient demographics and clinical data are described in Table 1. A first group consisted of six women who were vaccinated against SARS-CoV-2 infection (three with Pfizer-BioNTech COVID-19 vaccine, two with Oxford/AstraZeneca vaccine, and one with Moderna vaccine). The median times from the second vaccine dose to recruitment and sampling were 29 days (range 18–55 days). A second group consisted of five women who were SARS-CoV-2 affected but became fully negative (as confirmed by a molecular test) at the time of IVF; their clinical mild symptoms are reported in Supplementary Table 1. The average time between the infection of the patients with SARS-CoV-2 and the retrieval of FF was 7 months (interval: 4–11 months). Both groups were selected from March to September 2021. Nine healthy women were used as the control group, they were selected prior to the start of coronavirus pandemic, from June to December 2019.

**TABLE 1:** Clinical data of the 20 women participating in the study.

	SARS-Cov-2 vaccinated	Recovered COVID-19	Control^§^
*N*. patients	6	5	9
Age (years)	36.2 (4.3)	37.4 (5.9)	36.2 (4.2)
FSH (UI/ml)	6.7 (2.6)	6.5 (3.0)	6.8 (2.0)
AMH (ng/mL)	3.1 (3.1)	2.2 (1.2)	4.3 (4.6)
AFC	11.5 (5.2)	11.4 (5.2)	13.3 (4.2)
Estradiol (pg/mL)	1,828.0 (1,183.5)	1,195.1 (606.2)	1,848.6 (1232.1)
Progesterone (ng/mL)	1.3 (0.9)	1.2 (0.4)	1.3 (0.9)
BMI (kg/m^2^)	23.9 (4.1)	24.9 (5.3)	22.4 (3.5)
Follicles monitored	8.3 (2.9)	7.0 (4.6)	11.7 (4.7)
Total oocytes collected	4.8 (1.9)	3.8 (3.1)	9.2 (6.1)
MII oocytes	3.2 (1.0)	3.2 (2.3)	7.0 (5.3)
Zygotes	2.5 (1.4)	2.4 (1.7)	1.6 (0.7)*
Blastocysts	1.5 (1.4)	1.0 (0.7)	1.6 (0.7)*

*Data are presented as mean values and standard deviation are reported in parentheses.*

*^§^ Healthy pre-COVID.*

*FSH, follicle stimulating hormone; AMH, anti-Mullerian hormone; AFC, antral follicle count; BMI, body mass index.*

*The average number of zygotes and blastocysts was calculated on eight healthy controls because two MII oocytes were cryopreserved for one woman.

The infertility indications for the group of vaccinated women were: four male infertility factor, one unexplained infertility, and one tubaric disease; for the group of recovered COVID-19 patients the infertility indications were: two male infertility factor, two unexplained infertility, and one tubaric disease. All the control women presented mild or moderate male infertility factor.

Written informed consent was obtained from all participants enrolled in the study that was approved by the local ethical committee (Comitato Etico Unico Regionale per la Basilicata, approval number: 82/2015 of October 7, 2015).

All patients performed ovarian reserve tests: basal follicle-stimulating hormone (FSH), anti-mullerian hormone (AMH), and antral follicle count (AFC) before starting ovarian stimulation to have homogeneous samples with respect to the ovarian reserve.

Participants received stimulation with recombinant FSH (Gonal-f, Merck Serono, Roma, Italy or Ovaleap, Theramex, Milano, Italy) or urinary highly purified FSH (Fostimon, IBSA Farmaceutici Lodi, Italy) and gonadotropin-releasing hormone (GnRH) antagonist (Cetrotide, Merk Serono or Fyremadel, Ferring, Milano, Italy). In particular, follicular stimulation was started on day 2 of menstrual cycle with an FSH dose calculated according to the nomogram of La Marca et al. (2013). Follicular growth was monitored with ultrasound scans and estradiol and progesterone assessment, first on day 5 and then every 2 days. Daily administration of a GnRH antagonist (0.25 mg of Cetrotide or 0.25 mg of Fyremadel) was started when the leading follicle was 14 mm in diameter and continued until the day of ovulation trigger.

When at least two follicles had reached 17–18 mm in diameter, ovulation was triggered with a single subcutaneous bolus of 10.000 IU of highly purified human chorionic gonadotropin (hCG) (Gonasi HP 10.000, IBSA). The oocytes retrieval was performed after 34–36 h. The collection of cumulus-oocyte complexes and FFs were performed via transvaginal ultrasound-guided aspiration with a needle of 18 gauge of diameter. The three major diameter follicles were identified by intraoperative ultrasound and FF of each follicle was collected in its own test tube. We excluded FFs containing blood to avoid altering metabolomics analysis and we removed FF containing more than one oocyte.

The oocytes were placed in a buffered medium (G-MOPS Plus, Vitrolife) at 37°C and subsequently incubated at 37°C and 6% of CO2 in a medium containing bicarbonate, gentamicin, and human albumin (G-IVF, Vitrolife, Göteborg, Sweden) until the moment of decoronization. After 2–4 h of incubation, the *in vitro* cumulus and corona radiata cells were removed by hyaluronidase treatment and pipetting.

This procedure was adopted for all oocytes subjected to intracytoplasmic sperm injection (ICSI). To prevent the fertilization technique from affecting the ART outcomes, we fertilized all oocytes with ICSI. Embryos were transferred into the uterus only on the 5th day, under ultrasound guidance, using soft or rigid catheters. Blastocysts were deposited at 1.5 cm from the uterus fundus.

### Nuclear Magnetic Resonance Spectroscopy Sample Analysis

The aspirated FF was centrifuged at 10,000 rpm for 10 min to remove erythrocytes and leukocytes. The supernatant was collected and maintained frozen at −80°C until processing. Only FF samples not contaminated by the flushing medium during the aspiration procedure were used in the analysis.

^1^H NMR spectroscopy was performed as previously reported (Castiglione Morelli et al., 2018, 2019), here briefly described.

Samples were defrosted at room temperature before using. 600 μl of the supernatant was mixed with 58 μl of D_2_O and 5 μl of TSP (3-trimethylsilyl propionic acid-d_4_ sodium salt), that was used as chemical shift reference (δ = 0). All spectra were acquired at 25°C on a Varian 500 MHz spectrometer with no sample rotation; a Carr-Purcell-Meiboom-Gill (CPMG) pulse sequence was used to suppress the signals originating from macromolecules, with a 136 ms total spin echo time. A pre-saturation of the water peak was used. A total of 128 scans and 16K points were acquired with a spectral width of 5,995 Hz and a recycle delay of 5 s. The spectra were Fourier transformed with FT size of 32K and a 1 Hz line-broadening, phased and a polynomial baseline correction was applied over the whole spectral range.

The software advanced chemistry development (ACD)/1D NMR Processor (Academic Edition, ACD Labs, Toronto, ON, Canada) was used for processing all the spectra and producing integral buckets of 0.04 ppm. The TSP signal and the region 4.7--5.1 ppm, around water signal, were excluded. The integrated region was normalized to the total spectrum area. Metabolites responsible for sample differentiation were identified using data from literature or from the data banks human metabolome database (HMDB)^1^ and biological magnetic resonance banc (BMRB).^2^

### Multivariate Analysis

Nuclear Magnetic Resonance Spectroscopy data were imported into the program SIMPCA-P + (version 12, Umetrics, Umeå, Sweden) and subjected to pre-treatment with Pareto scaling (/√SD) which automatically mean-centers the data. An unsupervised Principal Component Analysis (PCA) model was built on the entire data set.

Projection to Latent Structures regression (PLS) was applied to build supervised models using different groups of women: a first group of six participants with vaccination against SARS-CoV-2 infection; a second group consisted of five recovered COVID-19 patients, and the third group consisted of nine healthy participants who were examined before COVID-19 pandemic. The overall quality of the models obtained by PLS discriminant analysis (PLS-DA) was evaluated by the *R*^2^ and *Q*^2^ values, where *R*^2^ measures the goodness of fit and displays the explained variation by components and *Q*^2^ gives an indication of the goodness of predicted model. The PLS-DA models were validated using permutation tests.

**FIGURE 1:**
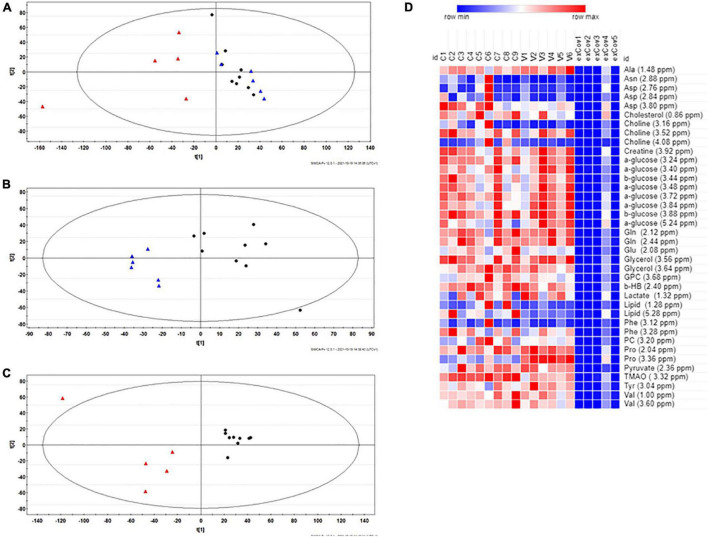
**(A)** Principal Component Analysis (PCA) score plot obtained from the ^1^H-NMR follicular fluid (FF) spectral data of all the 20 women examined in this study. **(B)** PLS discriminant analysis (PLS-DA) score plot between healthy women and vaccinated women. The *R*^2^X and *Q*^2^ values for the two-component model were: 0.47 and 0.56, respectively; **(C)** PLS-DA score plot between healthy women and COVID-19 recovered patients. The *R*^2^X and *Q*^2^ values for the two-component model were: 0.51 and 0.72, respectively. Data were colored by group: healthy control (*N* = 9, black dots), vaccinated (*N* = 6, blue triangles) and recovered COVID-19 (*N* = 5, red triangles). **(D)** Heat map of the most relevant metabolites (with a VIP value > 1) that were associated with the differentiation between healthy (*N* = 9, group C) and vaccinated (*N* = 6, group V) and recovered COVID-19 women (*N* = 5, exCov). Rows: quantification of NMR integral bin regions of metabolites with VIP > 1. Columns: different groups of women. Color scale indicates values ranging from blue (the lowest) to red (the highest).

The heatmap shown in Figure 1D was calculated with Morpheus software.^3^

### Follicular Fluid Anti-coronavirus Disease 2019 Immunoglobulins Detection

For the qualitative detection of the presence of anti-SARS-COV-2 IgG/IgM in 10 μl of FF, the nCOVID-19IgG&IgM POCT kit (Technogenetics, Milano, Italy) was used in accordance with the manufacturer’s instructions. The kit adopts the solid phase colloidal gold immunochromatographic technology.

### Follicular Fluid Western Blot Analysis

Lysates from follicular fluid were prepared with radioimmunoprecipitation assay (RIPA) buffer (50 mM Tris–HCl pH 7.5, 150 mM NaCl, 1% Non-idet P-40, 0.5% sodium deoxycholic acid) and 1% protease inhibitor cocktail (Sigma, Saint Louis, MO, United States). A total of 5 μg protein from each sample was separated by 15% SDS-PAGE and electrotransfered to a nitrocellulose membrane (Amersham Bioscience, Buckinghamshire, United Kingdom). Membranes were stained with Ponceau S solutions (Sigma, Saint Louis, MO, United States) for 15 min on the shaker at room temperature and then rinsed with distilled water to remove background stain for 5 min. Membranes were blocked with 5% milk for 1 h at room temperature and incubated overnight at 4°C using the following primary antibodies: 1:100 Anti-SOD2 (sc-130345) (Santa Cruz Biotechnology, Inc., Dallas, TX, United States), 1:200 Anti-IL-6 Antibody (E-4) (sc-28343) (Santa Cruz Biotechnology, Inc., Dallas, TX, United States), and 1:400 Anti-TNFα Antibody (C-4) (sc-133192) (Santa Cruz Biotechnology, Inc., Dallas, TX, United States). Membranes were subsequently incubated with horseradish peroxidase-conjugated secondary antibody [1:2,500 Anti-Mouse IgG (Fab specific)-Peroxidase antibody produced in goat (A9917) Sigma, Saint Louis, MO, United States] for 1 h at room temperature and then evaluated with an ECL™ Western Blotting Detection Reagents (Amersham Bioscience, Buckinghamshire, United Kingdom). Images were captured with Chemidoc™ XRS detection system equipped with Image Lab Software for image acquisition (BioRad) and processed using GelAnalizer 2010 software (Istvan Lazar^4^). Band intensities were totaled for each lane. Normalization using Ponceau S stain was conducted to minimize any discrepancies in protein amount. All experiments were replicated three times.

### Statistical Analysis

Normally distributed clinical data were compared across study groups by univariate ANOVA (Systat 11.0, Systat Software, Inc., San Jose, CA, United States). Pairwise comparisons of the means were performed with Fisher’s least significant differences (LSD) test. The minimum level of statistical significance was *p* < 0.05. Values are presented as mean ± standard deviation (SD).

## Results

The cohort of women examined consisted of 20 women. They were selected as follows: six women who received vaccination against SARS-CoV-2 infection, five recovered COVID-19 patients, and as a control, we chose nine women whose FFs were collected before the coronavirus pandemic outbreak. Patient’s demographics and steroidogenic characteristics are reported in Table 1. There were no significant differences in these parameters when comparing vaccinated women, recovered COVID-19 patients, and healthy controls.

We found that the normalized ratio of serum estradiol/oocytes changes among the groups being 380.9, 314.5, and 200.9, for vaccinated women, previously positive patients, and controls, respectively. Indeed, in both vaccinated women and recovered COVID-19 patients the oocytes retrieved/trigger day mature follicle count was 0.6 and 0.8 in healthy controls, respectively; the oocytes retrieved/serum progesterone in vaccinated women was 3.7 vs. 3.2 and 7.1 in recovered COVID-19 patients and controls, respectively. Finally, in vaccinated women the ratio of mature/total number of aspirated oocytes was 0.7 vs. 0.8 of both previously positive patients and controls, respectively. The average number of zygotes in vaccinated women (2.5 ± 1.4) and recovered COVID-19 patients (2.4 ± 1.7) is higher than in healthy controls (1.6 ± 0.7), although the difference was not significant. However, the number of blastocysts for the healthy controls remains the same of zygotes while it decreases in both vaccinated and ex-COVID-19 women (*p* > 0.05).

### Nuclear Magnetic Resonance Spectroscopy Results

An exploratory analysis of the ^1^H NMR spectra from all FFs was first made using PCA to reveal the main trends in the data set. A three-component model was obtained with cumulative *R*^2^ and *Q*^2^ values of 0.66 and 0.06, respectively, and the PCA scores plot reported in Figure 1A shows a quite clear clustering of the data and gives evidence of metabolic differences between the groups, with the vaccinated women being closer to healthy controls than the recovered COVID-19 patients.

These differences observed by unsupervised analysis were confirmed by PLS-DA. The calculated two-component model reported in Supplementary Figure 1 shows that the first two principal components contributed positively to discriminate the healthy women and the groups formed by vaccinated and previously positive women. To have further confirmation of the discrimination, we investigated the effects on FF of vaccination against SARS-CoV-2 or COVID-19 infection by pair-wise comparisons of these classes respect to healthy controls by using PLS-DA. The results are shown in Figures 1B,C, respectively.

Furthermore, PLS-DA generated a list of 39 signals with Variable Importance in the Projection (VIP) values > 1, which represent 21 potential metabolites useful for the discrimination of the three groups (Supplementary Table 2). Four metabolites resulted significantly different in the group of vaccinated women in comparison to healthy controls: the levels of Ala and Pro were higher while those of lipids and trimethylamine N-oxide (TMAO) were lower (Figure 1D). On the other hand, in the group of recovered COVID-19 patients, there are significant lower levels of aminoacids (Ala, Glu, Gln, Phe, Tyr, and Val), membrane components (cholesterol, choline, glycerol, glycerophosphocholine, and phosphocholine), and other metabolites (β-hydroxybutyrate, glucose, pyruvate, and TMAO).

Although the number of subjects included in this study is limited, our results suggest that in comparison to healthy pre-COVID subjects the FFs of previously infected women display more metabolic alterations than those of vaccinated women.

### Immunoglobulin Measurements

Only recovered COVID-19 patients showed specific IgG antibodies in follicular fluid, that were absent both in vaccinated and control subjects (Supplementary Figure 2).

### Evaluation of Some Oocyte Quality Biomarkers

**FIGURE 2:**
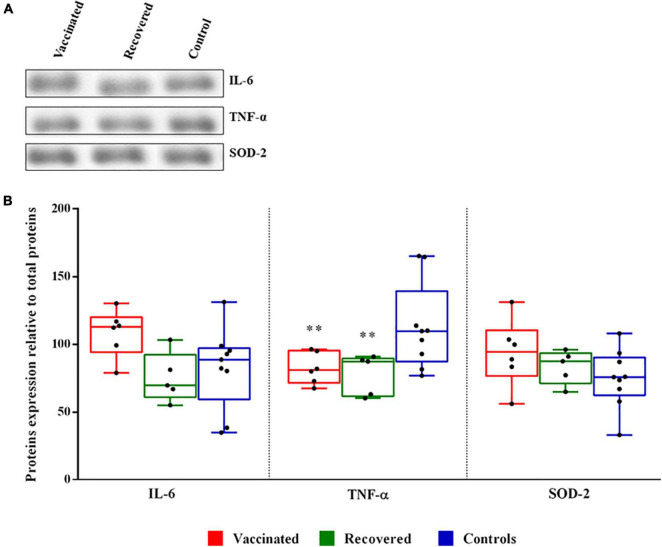
Expression level of interleukin-6 (IL-6), tumor necrosis factor-α (TNF-α), and superoxide dismutase 2 (SOD-2) proteins in follicular fluids. **(A)** Representative western blot of proteins in follicular fluid (FF). **(B)** Densitometric analysis of the immunoreactive bands performed in three independent experiments. After densitometric analysis, western blot signals of the target proteins are normalized to the total amount of protein in each lane. The box plots show medians and whiskers. Significance (^**^p < 0.01).

The analysis of both cytokines and sensors of altered reactive oxygen species (ROS) levels, is crucial to appreciate the role of FF milieu on follicle development. To determine whether SARS-CoV-2 infection and vaccines alter the expression of inflammation-associated proteins, we evaluated by western blot analysis the expression levels of proinflammatory cytokines TNF-α and IL-6. Moreover, the expression level of mitochondrial superoxide dismutase SOD-2, a sensor of redox balance, was also evaluated (Figure 2A). Statistical analysis revealed no significant difference between IL-6 and SOD-2 in follicular fluids derived from recovered COVID-19 patients and vaccinated women compared to healthy controls women. Interestingly, median expression level of TNF-α is significantly lower in recovered COVID-19 patients and vaccinated compared to healthy controls women (Figure 2B).

## Discussion

Anti-Mullerian hormone (AMH) and antral follicle count (AFC) together are defined as two valid biomarkers in the evaluation of ovarian reserve and patient response to hormonal therapies (Broer et al., 2011). No significant difference was observed between vaccinated women, previously positive patients, and controls (Table 1).

Several studies have reported that the E2/oocytes ratio is an important predictive parameter for IVF success (Bu et al., 2015; Taheri et al., 2020). However, Aslih et al. (2021) have demonstrated that the E2/M2 ratio is a better biomarker and that lower ratios produced higher quality embryos and a better pregnancy rate. We found a higher E2/M2 ratio in both vaccinated and recovered women (571 and 373, respectively) in comparison to the control one (264).

Ultimately, even if there is no statistically significant difference between the three groups of women considered, the clinical data seem to indicate some difference between them. In particular, the adequacy of the follicular response to ovulation triggering tends to decrease in vaccinated women and recovered COVID-19 patients compared to controls.

In this study we have found some metabolic differences in FFs of vaccinated women and previously infected patients in comparison with healthy controls. In particular, for vaccinated women we found higher levels of the amino acids Ala and Pro and lower levels of lipids and TMAO. TMAO is a metabolite produced from dietary choline and carnitine by the gut microbiota and, successively, by hepatic flavin monooxygenases. In a recent study Nagy et al. (2020) showed that TMAO is associated with unfavorable fertility outcomes: TMAO is significantly lower in FF from oocytes that underwent normal fertilization and developed into top-quality embryos. Our finding of lower levels of TMAO in FFs of vaccinated women, if confirmed on a higher number of cases, could then be reassuring about the fertility outcomes of these women undergoing ART treatment.

We showed that FFs of recovered patients were positive for IgG antibodies against SARS-CoV-2, as found elsewhere (Herrero et al., 2022). No detectable levels of anti-COVID IgG in follicular fluid of vaccinated women were found.

Besides, lower levels of many important metabolites were found in recovered COVID patients’ FFs together with the lowest number of retrieved oocytes; this last finding was also reported by Herrero et al. (2022).

Oxidative stress, referred to an imbalance between the concentrations of pro- and anti-oxidants, may play a role during folliculogenesis and oogenesis (Da Broi et al., 2018). Granulosa and cumulus cells protect the oocyte from oxidative stress damage also regulating the expression of the various SOD isoforms (von Mengden et al., 2020). SOD-2 in human follicular fluid represents a biomarker of follicle and in turn oocyte quality. Although not significantly, SOD-2 levels in FF of vaccinated and recovered women tend to be higher than in controls to indicate that cells respond with an increase in defense mechanisms to counterbalance a probable imbalance of redox homeostasis, thus preventing impairment of the oocyte quality.

Physiological expression of cytokines and chemokines is required for optimal ovarian functions; they are produced by resident and infiltrating leukocytes as well as granulosa and theca cells (Akison et al., 2018). Inflammatory processes might compromise steroidogenesis and oocyte development; in particular, it has been observed that pro-inflammatory cytokines in FF play essential roles in follicular growth and ovulation, influence subsequent fertilization, embryonic development, implantation potential, and implantation of embryos (Adamczak et al., 2021). It has been shown that patients with severe COVID-19 disease can be subjected to the so-called “cytokine storm” (Chen et al., 2021), an exaggeratedly violent reaction of the immune defenses which, instead of protecting from the virus, attack all the patient’s organs. Plasma levels of some inflammatory mediators such as interferon-gamma, TNF-α and IL-6 were also particularly high (Cabaro et al., 2021; Coperchini et al., 2021). Consequently, variations in the plasma composition could affect the cytokines composition of FF. In both vaccinated and recovered women, no significant variation in the levels of the two proinflammatory cytokines was observed, compared to those of controls. Even, the level of TNF-α expression is significantly reduced, indicating that the quality of the oocytes should not be compromised by inflammatory events attributable to vaccination and mild COVID-19 infection. Furthermore, in agreement with low expression of TNF-α we also found a lower level of TMAO to confirm a reduced inflammatory state (Yang et al., 2019).

All considered, our results do not indicate high oxidative stress and inflammation in FFs of both the vaccinated and recovered women, compared to controls and therefore neither the folliculogenesis nor the quality of the oocytes would seem to be compromised.

Our study has two main limitations, that is the small number of women examined and the different time interval passed between infection or vaccination and oocyte collection, that did not consider properly the time-dependency of the effect of immune response induced by infection or vaccination. Further studies should then be performed with a larger cohort of women and potential long-term effects of vaccination and COVID-19 infection on fertility should be also evaluated.

## Data Availability Statement

The original contributions presented in the study are included in the article/Supplementary Material, further inquiries can be directed to the corresponding author.

## Ethics Statement

The studies involving human participants were reviewed and approved by Comitato Etico Unico Regionale per la Basilicata, approval number: 82/2015 of October 7, 2015. The patients/participants provided their written informed consent to participate in this study.

## Author Contributions

SS, AI, and AO designed the study. AI selected the patients and executed oocyte retrieval. AF identified oocytes in follicular fluid and executed their fertilization. PC selected the follicular fluids to be used for metabolomic analyses. MC performed the analysis of NMR data and multivariate analysis. LV run the NMR experiments. IM performed western blotting analyses and Ig search. AO, AI, and MC were responsible for conducting the study and wrote the manuscript which was critically discussed, edited, and approved by all co-authors. All authors contributed to the article and approved the submitted version.

## Conflict of Interest

The authors declare that the research was conducted in the absence of any commercial or financial relationships that could be construed as a potential conflict of interest.

## Publisher’s Note

All claims expressed in this article are solely those of the authors and do not necessarily represent those of their affiliated organizations, or those of the publisher, the editors and the reviewers. Any product that may be evaluated in this article, or claim that may be made by its manufacturer, is not guaranteed or endorsed by the publisher.

## Footnotes

1
http://www.hmdb.ca/

2
http://www.bmrb.wisc.edu/metabolomics/

3
https://software.broadinstitute.org/morpheus

4
www.gelanalyzer.com

## References

[B1] Adamczak R. Ukleja-Sokołowska N. Lis K. Dubiel M. (2021). Function of follicular cytokines: roles played during maturation, development and implantation of embryo. *Medicina* 57:1251. 10.3390/medicina57111251 34833469 PMC8625323

[B2] Akison L. K. Robertson S. A. Gonzalez M. B. Richards J. S. Smith C. W. Russell D. L. (2018). Regulation of the ovarian inflammatory response at ovulation by nuclear progesterone receptor. *Am. J. Reprod. Immunol.* 79:e12835. 10.1111/aji.12835 29484756

[B3] Alviggi C. Esteves S. C. Orvieto R. Conforti A. La Marca A. Fischer R. (2020). POSEIDON (Patient-Oriented Strategies Encompassing IndividualizeD Oocyte Number) group. COVID-19 and assisted reproductive technology services: repercussions for patients and proposal for individualized clinical management. *Reprod. Biol. Endocrinol.* 18:45. 10.1186/s12958-020-00605-z 32404170 PMC7218705

[B4] Andrabi S. W. Jaffar M. Arora P. R. (2020). COVID-19: new adaptation for IVF laboratory protocols. *JBRA Assist. Reprod.* 24 358–361. 10.5935/1518-0557.20200054 32598835 PMC7365532

[B5] Asampille G. Cheredath A. Joseph D. Adiga S. K. Atreya H. S. (2020). The utility of nuclear magnetic resonance spectroscopy in assisted reproduction. *Open Biol.* 10:200092. 10.1098/rsob.200092 33142083 PMC7729034

[B6] Aslih N. Michaeli M. Mashenko D. Ellenboge A. Lebovitz O. Atzmon Y. (2021). More is not always better-lower estradiol to mature oocyte ratio improved IVF outcomes. *Endocr. Connect.* 10 146–153. 10.1530/EC-20-0435 33416511 PMC7983485

[B7] Baskind N. E. McRae C. Sharma V. Fisher J. (2011). Understanding subfertility at a molecular level in the female through the application of nuclear magnetic resonance (NMR) spectroscopy. *Hum. Reprod. Update* 17 228–241. 10.1093/humupd/dmq039 20801939

[B8] Bentov Y. Beharier O. Moav-Zafrir A. Kabessa M. Godin M. Greenfield C. S. (2021). Ovarian follicular function is not altered by SARS-CoV-2 infection or BNT162b2 mRNA COVID-19 vaccination. *Hum. Reprod.* 36 2506–2513. 10.1093/humrep/deab182 34364311 PMC8385874

[B9] Bianchi L. Gagliardi A. Landi C. Focarelli R. De Leo V. Luddi A. (2016). Protein pathways working in human follicular fluid: the future for tailored IVF? *Expert Rev. Mol. Med.* 18:e9. 10.1017/erm.2016.4 27149979

[B10] Broer S. L. Dólleman M. Opmeer B. C. Fauser B. C. Mol B. W. Broekmans F. J. (2011). AMH and AFC as predictors of excessive response in controlled ovarian hyperstimulation: a meta-analysis. *Hum. Reprod. Update* 17 46–54. 10.1093/humupd/dmq034 20667894

[B11] Bruzzone C. Bizkarguenaga M. Gil-Redondo R. Diercks T. Arana E. García de Vicuña A. (2020). SARS-CoV-2 infection dysregulates the metabolomic and lipidomic profiles of serum. *iScience* 23:101645. 10.1016/j.isci.2020.101645 33043283 PMC7534591

[B12] Bu Z. Wang K. Guo Y. Su Y. Zhai J. Sun Y. (2015). Impact of estrogen-to-oocyte ratio on live birth rate in women undergoing in vitro fertilization and embryo transfer. *Int. J. Clin. Exp. Med.* 8 11327–11331. 26379944 PMC4565327

[B13] Cabaro S. D’Esposito V. Di Matola T. Sale S. Cennamo M. Terracciano D. (2021). Cytokine signature and COVID-19 prediction models in the two waves of pandemics. *Sci. Rep.* 11:20793. 10.1038/s41598-021-00190-0 34675240 PMC8531346

[B14] Castiglione Morelli M. A. Iuliano A. Schettini S. C. A. Petruzzi D. Ferri A. Colucci P. (2018). NMR metabolomics study of follicular fluid in women with cancer resorting to fertility preservation. *J. Assist. Reprod. Genet.* 35 2063–2070. 10.1007/s10815-018-1281-7 30069850 PMC6240554

[B15] Castiglione Morelli M. A. Iuliano A. Schettini S. C. A. Petruzzi D. Ferri A. Colucci P. (2019). NMR metabolic profiling of follicular fluid for investigating the different causes of female infertility: a pilot study. *Metabolomics* 15:19. 10.1007/s11306-019-1481-x 30830455

[B16] Chen R. Lan Z. Ye J. Pang L. Liu Y. Wu W. (2021). Cytokine storm: the primary determinant for the pathophysiological evolution of COVID-19 deterioration. *Front. Immunol.* 12:589095. 10.3389/fimmu.2021.589095 33995341 PMC8115911

[B17] Chen Y. M. Zheng Y. Yu Y. Wang Y. Huang Q. Qian F. (2020). Blood molecular markers associated with COVID-19 immunopathology and multi-organ damage. *EMBO J.* 39:e105896. 10.15252/embj.2020105896 33140861 PMC7737620

[B18] Coperchini F. Chiovato L. Rotondi M. (2021). Interleukin-6, CXCL10 and infiltrating macrophages in COVID-19-related cytokine storm: not one for all but all for one! *Front. Immunol.* 12:668507. 10.3389/fimmu.2021.668507 33981314 PMC8107352

[B19] Costa Dos Santos Junior G. Pereira C. M. Kelly da Silva Fidalgo T. Valente A. P. (2020). Saliva NMR-based metabolomics in the war against COVID-19. *Anal. Chem.* 92 15688–15692. 10.1021/acs.analchem.0c04679 33215503

[B20] Da Broi M. G. Giorgi V. S. I. Wang F. Keefe D. L. Albertini D. Navarro P. A. (2018). Influence of follicular fluid and cumulus cells on oocyte quality: clinical implications. *J. Assist. Reprod. Genet.* 35 735–751. 10.1007/s10815-018-1143-3 29497954 PMC5984887

[B21] Demirel C. Tulek F. Celik H. G. Donmez E. Tuysuz G. Gökcan B. (2021). Failure to detect viral RNA in follicular fluid aspirates from a SARS-CoV-2-positive woman. *Reprod. Sci.* 28 2144–2146. 10.1007/s43032-021-00502-9 33616884 PMC7899067

[B22] Doğan H. O. Şenol O. Bolat S. Yıldız ŞN. Büyüktuna S. A. Sarıismailoğlu R. (2021). Understanding the pathophysiological changes via untargeted metabolomics in COVID-19 patients. *J. Med. Virol.* 93 2340–2349. 10.1002/jmv.26716 33300133

[B23] Elsaddig M. Khalil A. (2021). Effects of the COVID pandemic on pregnancy outcomes. *Best Pract. Res. Clin. Obstet. Gynaecol.* 73 125–136. 10.1016/j.bpobgyn.2021.03.004 33832868 PMC7969862

[B24] Field S. L. Dasgupta T. Cummings M. Orsi N. M. (2014). Cytokines in ovarian folliculogenesis, oocyte maturation and luteinisation. *Mol. Reprod. Dev.* 81 284–314. 10.1002/mrd.22285 24273059

[B25] Fraser D. D. Slessarev M. Martin C. M. Daley M. Patel M. A. Miller M. R. (2020). Metabolomics profiling of critically Ill coronavirus disease 2019 patients: identification of diagnostic and prognostic biomarkers. *Crit. Care Explor.* 2:e0272. 10.1097/CCE.0000000000000272 33134953 PMC7587450

[B26] Freitas C. Neto A. C. Matos L. Silva E. Ribeiro Â Silva-Carvalho J. L. (2017). Follicular fluid redox involvement for ovarian follicle growth. *J. Ovarian Res.* 10:44. 10.1186/s13048-017-0342-3 28701210 PMC5508613

[B27] Herrero Y. Pascuali N. Velázquez C. Oubña G. Hauk V. de Zúñiga I. (2022). SARS-CoV-2 infection negatively affects ovarian function in ART patients. *Biochim. Biophys. Acta Mol. Basis Dis.* 1868:166295. 10.1016/j.bbadis.2021.166295 34718118 PMC8550892

[B28] Kassie A. Wale A. Yismaw W. (2021). Impact of coronavirus diseases-2019 (COVID-19) on utilization and outcome of reproductive, maternal, and newborn health services at governmental health facilities in South West Ethiopia, 2020: comparative cross-sectional study. *Int. J. Womens Health* 13 479–488. 10.2147/IJWH.S309096 34040456 PMC8141395

[B29] Kimhofer T. Lodge S. Whiley L. Gray N. Loo R. L. Lawler N. G. (2020). Integrative modeling of quantitative plasma lipoprotein, metabolic, and amino acid data reveals a multiorgan pathological signature of SARS-CoV-2 infection. *J. Proteome Res.* 19 4442–4454. 10.1021/acs.jproteome.0c00519 32806897

[B30] Kteily K. Pening D. Vidal P. D. Devos M. Dechene J. Op De Beeck A. (2021). Risk of contamination of semen, vaginal secretions, follicular fluid and ovarian medulla with SARS-CoV-2 in patients undergoing ART. *Hum. Reprod.* 37, 235–241. 10.1093/humrep/deab255 34741508 PMC8689924

[B31] La Marca A. Grisendi V. Giulini S. Argento C. Tirelli A. Dondi G. (2013). Individualization of the FSH starting dose in IVF/ICSI cycles using the antral follicle count. *J. Ovarian Res.* 6:11. 10.1186/1757-2215-6-11 23388048 PMC3568720

[B32] Lodge S. Nitschke P. Kimhofer T. Coudert J. D. Begum S. Bong S. H. (2021). NMR spectroscopic windows on the systemic effects of SARS-CoV-2 infection on plasma lipoproteins and metabolites in relation to circulating cytokines. *J. Proteome Res.* 20 1382–1396. 10.1021/acs.jproteome.0c00876 33426894

[B33] Madjunkov M. Dviri M. Librach C. (2020). A comprehensive review of the impact of COVID-19 on human reproductive biology, assisted reproduction care and pregnancy: a Canadian perspective. *J. Ovarian Res.* 13:140. 10.1186/s13048-020-00737-1 33246480 PMC7694590

[B34] McRae C. Baskind N. E. Orsi N. M. Sharma V. Fisher J. (2012). Metabolic profiling of follicular fluid and plasma from natural cycle in vitro fertilization patients–a pilot study. *Fertil. Steril.* 98 1449–1457.e6. 10.1016/j.fertnstert.2012.07.1131 22921074

[B35] Nagy R. A. Homminga I. Jia C. Liu F. Anderson J. L. C. Hoek A. (2020). Trimethylamine-N-oxide is present in human follicular fluid and is a negative predictor of embryo quality. *Hum. Reprod.* 35 81–88. 10.1093/humrep/dez224 31916569 PMC9185935

[B36] O’Gorman A. Wallace M. Cottell E. Gibney M. J. McAuliffe F. M. Wingfield M. (2013). Metabolic profiling of human follicular fluid identifies potential biomarkers of oocyte developmental competence. *Reproduction* 146 389–395. 10.1530/REP-13-0184 23886995

[B37] Páez-Franco J. C. Torres-Ruiz J. Sosa-Hernández V. A. Cervantes-Díaz R. Romero-Ramírez S. Pérez-Fragoso A. (2021). Metabolomics analysis reveals a modified amino acid metabolism that correlates with altered oxygen homeostasis in COVID-19 patients. *Sci. Rep.* 11:6350. 10.1038/s41598-021-85788-0 33737694 PMC7973513

[B38] Pñero-Sagredo E. Nunes S. de los Santos M. J. Celda B. Esteve V. (2010). NMR metabolic profile of human follicular fluid. *NMR Biomed.* 23 485–495. 10.1002/nbm.1488 20336675

[B39] Rajput S. K. Khan S. A. Goheen B. B. Engelhorn H. J. Logsdon D. M. Grimm C. K. (2021a). Absence of SARS-CoV-2 (COVID-19 virus) within the IVF laboratory using strict patient screening and safety criteria. *Reprod. Biomed. Online* 42 1067–1074. 10.1016/j.rbmo.2021.03.005 33814309 PMC7937039

[B40] Rajput S. K. Logsdon D. M. Kile B. Engelhorn H. J. Goheen B. Khan S. (2021b). Human eggs, zygotes, and embryos express the receptor angiotensin 1-converting enzyme 2 and transmembrane serine protease 2 protein necessary for severe acute respiratory syndrome coronavirus 2 infection. *F S Sci.* 2 33–42. 10.1016/j.xfss.2020.12.005 33521687 PMC7831752

[B41] Robertson S. A. Chin P. Y. Femia J. G. Brown H. M. (2018). Embryotoxic cytokines-potential roles in embryo loss and fetal programming. *J. Reprod. Immunol.* 125 80–88. 10.1016/j.jri.2017.12.003 29306096

[B42] Sapkota D. Søland T. M. Galtung H. K. Sand L. P. Giannecchini S. To K. K. W. (2020). COVID-19 salivary signature: diagnostic and research opportunities. *J. Clin. Pathol.* 74, 344–349. 10.1136/jclinpath-2020-206834 32769214

[B43] Shen B. Yi X. Sun Y. Bi X. Du J. Zhang C. (2020). Proteomic and metabolomic characterization of COVID-19 patient sera. *Cell* 182 59–72.e15. 10.1016/j.cell.2020.05.032 32492406 PMC7254001

[B44] Shi D. Yan R. Lv L. Jiang H. Lu Y. Sheng J. (2021). The serum metabolome of COVID-19 patients is distinctive and predictive. *Metabolism* 118:154739. 10.1016/j.metabol.2021.154739 33662365 PMC7920809

[B45] Shy J. Fan J. Su Q. Yang Z. (2019). Cytokines and abnormal glucose and lipid metabolism. *Front. Endocrinol.* 10:703. 10.3389/fendo.2019.00703 31736870 PMC6833922

[B46] Taheri F. Omidi M. Khalili M. A. Agha-Rahimi A. Sabour M. Faramarzi A. (2020). The determination of estradiol to Cumulus Oocyte Complex (COC) number ratio: does it predict the outcomes of ART cycles? *J. Reprod. Infertil.* 21 11–16. 32175261 PMC7048692

[B47] von Mengden L. Klamt F. Smitz J. (2020). Redox biology of human cumulus cells: basic concepts, impact on oocyte quality, and potential clinical use. *Antioxid. Redox Signal.* 32 522–535. 10.1089/ars.2019.7984 31861967 PMC7038817

[B48] Wallace M. Cottell E. Gibney M. J. McAuliffe F. M. Wingfield M. Brennan L. (2012). An investigation into the relationship between the metabolic profile of follicular fluid, oocyte developmental potential, and implantation outcome. *Fertil. Steril.* 97 1078–1084.E8. 10.1016/j.fertnstert.2012.01.122 22365382

[B49] Wu D. Shu T. Yang X. Song J.-X. Zhang M. Yao C. (2020). Plasma metabolomic and lipidomic alterations associated with COVID-19. *Natl. Sci. Rev.* 7 1157–1168. 10.1093/nsr/nwaa086 34676128 PMC7197563

[B50] Yang S. Li X. Yang F. Zhao R. Pan X. Liang J. (2019). Gut Microbiota-dependent marker TMAO in promoting cardiovascular disease: inflammation mechanism, clinical prognostic, and potential as a therapeutic target. *Front. Pharmacol.* 10:1360. 10.3389/fphar.2019.01360 31803054 PMC6877687

