# Supplementary material for: Are the Follicular Fluid Characteristics of Recovered Coronavirus Disease 2019 Patients Different From Those of Vaccinated Women Approaching in vitro Fertilization?
Source: Front Physiol. 2022 Feb 22;13:840109. doi: 10.3389/fphys.2022.840109 (PMC8905595; doi:10.3389/fphys.2022.840109)

Supplementary Material

Are the follicular fluid characteristics of recovered COVID-19 patients different from those of vaccinated women approaching in vitro fertilization?

Maria A. Castiglione Morelli,[1] Assunta Iuliano,[2] Sergio C. A. Schettini,[2] Angela Ferri,[2] Paola Colucci,[2] Licia Viggiani,[1] Ilenia Matera,[1] Angela Ostuni [1*]

[1] Department of Sciences, University of Basilicata, Potenza, Italy

[2] Center for Reproductive Medicine of "San Carlo" Hospital, Potenza, Italy

*** Correspondence:**
Angela Ostuni, Department of Sciences, University of Basilicata, viale Ateneo Lucano 10, 85100, Potenza, Italy (tel: 0039-0971-205453, email: angela.ostuni@unibas.it)

Supplementary Table 1 - Symptoms presented by SARS-CoV-2 positive patients before undergoing IVF treatments.

**Symptoms**	**SARS-CoV-2 patients (N =5)**
Fever	3
Arthralgia	2
Aphonia	1
Weakness	2
Odour-blindness	2

Supplementary Figure 1. PLS-DA score plot obtained from the ^{1}H-NMR FF spectral data of the 20 women examined. The R^2X, R^2Y and Q^2 values for this two-component model were 0.48, 0.69 and 0.56, respectively. Data were coloured by group: healthy control (N=9, black dots), vaccinated (N=6, blue triangles) and recovered COVID-19 (N=5, red triangles).

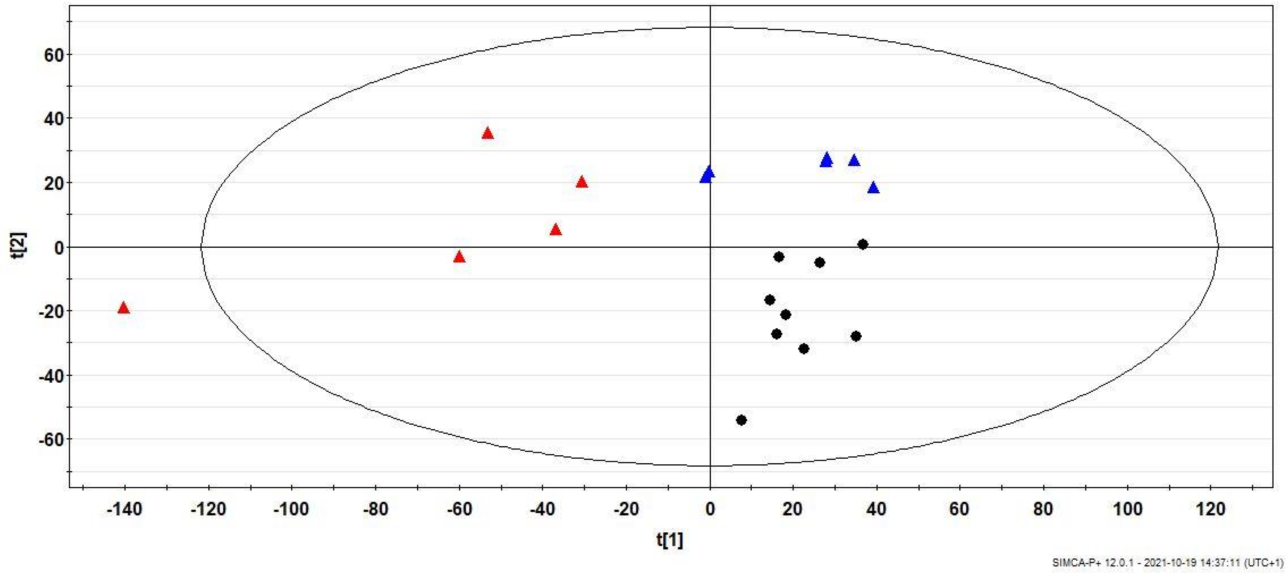

Supplementary Table 2. Metabolites identified by NMR which were useful to discriminate between follicular fluids of healthy control and women vaccinated against SARS-CoV-2 or recovered COVID-19 patients.

**Metabolite**	**NMR (δ)**	**Healthy (n=9)**	**Vaccinated (n=6)**	**VIP**	**p-value**	**Recovered (n=5)**	**VIP**	**p-value**
Ala	1.48	106.0 ± 17.4	130.8 ± 21.5	1.4	0.03	55.7 ± 25.9	1.4	0.0009
Asn	2.88	235.3 ± 266.2	63.7 ± 76.9	3.5	ns	256.7 ± 181.5	*	ns
Asp	3.80	296.4 ± 95.2	216.7 ± 76.3	2.4	ns	262.2 ± 95.1	*	ns
Asp	2.84	63.3 ± 59.7	30.9 ± 27.9	1.3	ns	132.5 ± 135.9	1.1	ns
Asp	2.76	66.8 ± 74.5	37.8 ± 24.4	1.5	ns	74.4 ± 76.5	*	ns
Cholesterol	0.88	119.5 ± 35.9	95.0 ± 30.1	1.1	ns	71.1 ± 44.2	1.2	0.04
Choline	3.16	235.9 ± 225.4	73.1 ± 61.6	3.5	ns	213.7 ± 155.9	*	ns
Choline	4.08	75.8 ± 71.5	30.6 ±7.9	1.7	ns	16.3 ± 11.6	1.3	ns
Choline	3.52	209.5 ± 53.5	196.7 ± 62.3	*	ns	103.9 ± 41.7	2.0	0.003
Creatine	3.92	237.5 ± 64.1	258.0 ± 68.1	*	ns	168.7 ± 61.9	1.4	ns
α-Glucose	3.84	215.9 ± 61.8	273.8 ±76.3	1.9	ns	168.1 ± 71.9	1.1	ns
α-Glucose	5.24	102.7 ± 43.8	140.8 ± 38.1	1.6	ns	129.7 ± 34.7	*	ns
β-Glucose	3. 40	142.2 ± 44.5	173.2 ± 47.1	1.2	ns	84.8 ± 35.9	1.6	0.006
α-Glucose	3.48	268.4 ± 83.8	306.2 ± 96.4	1.1	ns	149.6 ± 54.2	1.9	0.01
α-Glucose	3.72	285.1 ± 57.5	305.3 ± 81.9	1.0	ns	180.4 ± 87.2	1.8	0.02
α-Glucose	3.24	364.3 ± 68.9	367.1 ± 85.1	*	ns	165.7 ± 64.1	2.9	0.0002
β -Glucose	3.44	280.3 ± 75.1	269.8 ± 75.6	*	ns	124.4 ± 48.7	2.5	0.001
β -Glucose	3.88	176.3 ± 34.2	180.7 ± 39.8	*	ns	108.9 ± 44.6	1.5	0.008
Gln	2.12	96.2 ± 24.7	100.2 ± 14.4	*	ns	44.9 ± 15.4	1.4	0.001
Gln	2.44	76.8 ± 16.7	80.1 ± 14.0	*	ns	38.7 ± 12.7	1.3	0.0008
Glu	2.08	131.1 ± 41.5	101.6 ± 10.9	1.5	ns	58.0 ± 19.3	1.7	0.003
Glycerol	3.56	204.2 ± 50.1	216.7 ± 45.4	*	ns	99.0 ± 36.9	2.0	0.001
Glycerol	3.64	71.5 ± 14.5	63.8 ± 10.8	*	ns	29.4 ± 18.2	1.3	0.001
GPC	3.68	124.4 ± 37.5	105.9 ±18.9	*	ns	50.1 ± 34.4	1.7	0.01
β-HB	2.40	90.4 ± 16.8	79.5 ± 15.2	*	ns	45.2 ± 16.2	1.4	0.0004
Lactate	1.32	398.9 ± 208.4	539.3 ±186.5	2.7	ns	465.6 ± 220.9	*	ns
Lipid	1.28	171.7 ± 137.7	70.1 ±26.6	2.6	ns	57.2 ± 36.4	1.9	ns
Lipid	5.28	72.4 ± 33.3	29.9 ± 5.7	2.0	0.009	86.9 ± 49.9	*	ns
Phe	3.12	95.3 ± 104.4	51.8 ± 36.7	1.6	ns	100.7 ± 92.6	*	ns
Phe	3.28	217.3 ± 78.2	183.3 ± 51.9	1.3	ns	93.8 ± 26.1	2.1	0.006
PC	3.20	139.3 ± 66.5	129.7 ± 13.5	*	ns	59.1 ± 23.3	1.6	0.02
Pro	2.04	157.6 ± 33.3	177.9 ± 21.1	1.2	ns	90.3 ± 47.2	1.5	0.009
Pro	3.36	72. ± 23.3	192.4 ± 18.4	4.2	0.0000001	116.4 ± 68.3	1.5	ns
Pyruvate	2.36	66.3 ± 18.6	70.0 ± 12.2	*	ns	19.9 ± 8.7	1.5	0.0002
TMAO	3.32	48.2 ± 4.8	35.1 ± 5.6	1.2	0.0003	20.1 ± 11.4	1.1	0.00003
Tyr	3.04	54.6 ± 13.5	70.4 ± 14.9	1.1	ns	34.8 ± 16.1	*	0.03
Val	3.60	99.8 ± 21.2	88.7 ± 14.5	*	ns	40.2 ± 26.9	1.5	0.0006
Val	1.00	84.5 ± 19.3	81.4 ± 14.9	*	ns	46.4 ± 23.7	1.2	0.007

The average integrals of the NMR bin regions ± standard deviations are reported.
δ, NMR chemical shift
VIP, **V**ariable **I**mportance in the **P**rojection
*, VIP value < 1
P values were obtained by performing t-test between healthy controls and other women's groups. *ns* = p-value > 0.05
GPC, Glycerophosphocholine
β-HB, β-hydroxybutyrate
PC, Phosphocholine
TMAO, trimethylamine oxide

Supplementary Figure 2. Follicular Fluids anti-Covid-19 immunoglobulins measurements. (A) one vaccinated woman; (B) one recovered COVID-19 patient; visible purple bands appear both at the control line (C-line) and the test line 2 (T2) of the cassette; (C) one healthy control woman. In (A) and (C) a visible purple band appears only at the control line (C-line) of the cassette. For all the women IgM test line (T1) was negative.

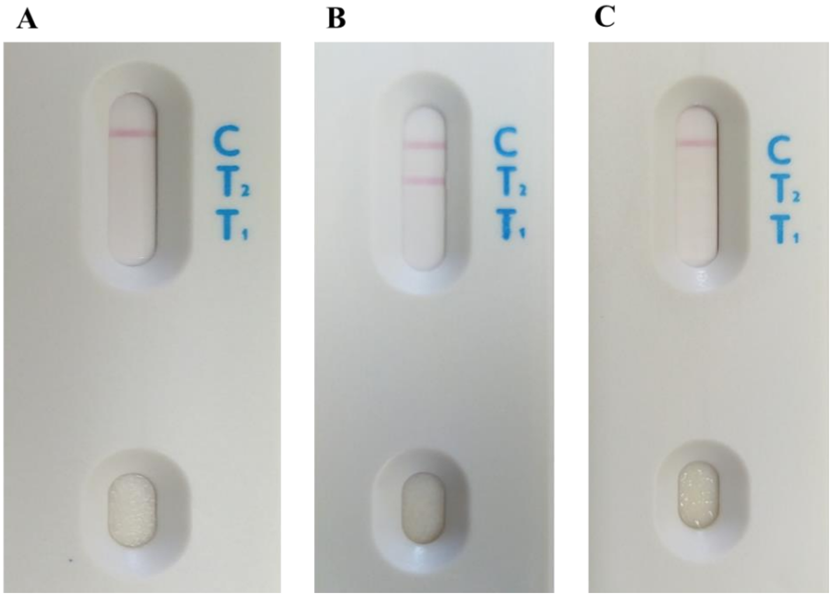

Supplement: Supplementary file 1 [file Data_Sheet_1.PDF]
